# Short, double elastic nailing of severely displaced distal pediatric radial fractures: A new method for stable fixation

**DOI:** 10.1097/MD.0000000000006532

**Published:** 2017-04-07

**Authors:** Marcell Varga, Gergő Józsa, Balázs Fadgyas, Tamás Kassai, Antal Renner

**Affiliations:** a Sándor Péterfy Street Hospital and Casualty Centre; b Surgical Department of Heim Pál Children's Hospital, Budapest; c Department of Pediatrics, Surgical Unit, University of Pécs, Pécs, Hungary.

**Keywords:** children, diametaphyseal radial fracture, distal forearm fracture, elastic nail

## Abstract

**Rationale:**

Short double elastic nailing is a minimal invasive, modified ESIN (elastic stable intramedullary nailing) technique for severely displaced distal radial fracture in children. The aim of this technical report is to introduce our new method and evaluate the final results of the procedure.

**Patient concerns:**

We reviewed retrospectively 24 patients who underwent short double elastic nailing due to distal radial fractures between November 2012 and December 2015. Indications for surgery included closed, severely displaced, unstable metaphyseal or diametaphyseal fractures of the radius.

**Intervention:**

The fractures were stabilized by 2 prebent short elastic titanium nails inserted from the distal side of the fracture. In cases of associated ulnar fracture, a classic anterograd ESIN nailing was also performed. Patients were mobilized immediately in a removable short splint which was removed after 1 to 2 weeks. There has been no additional splinting or casting.

**Outcomes:**

There were 17 males and 7 females with an average age of 9.8 years (range, 4–16 years). The right hand was involved in 16 cases and the left hand in 8 cases. The average follow-up was 17.8 months (range, 7–28 months). Of the 24 patients, 3 presented irritation of the skin, which resolved after removal of the radial nail. All the patients regained full range of motion without any complications.

**Lessons:**

Our technique is an effective, safe, and easily learnable procedure for unstable fractures of the distal third of the radius. It achieves good functional and radiological results, and allows early mobilization without the need of casting. Avoiding the physeal plates, we reduce the risk of iatrogenic postoperative deformity. Further prospective and biomechanical investigations are necessary to verify our experience.

## 1 Introduction

Distal and severely displaced pediatric metaphyseal radial fractures with total rupture of the periosteum are generally considered unstable.^[1]^ Although the remodeling potential of distal radius fractures is very good in childhood, a subgroup of severely displaced and unstable distal pediatric forearm fractures are candidates for operative fixation because acceptable reduction cannot be maintained in a conservative way.

These injuries are usually candidates for closed reduction and minimal invasive fixation.^[1–5]^ Operative osteosynthesis technique of pediatric wrist fractures is optimally minimally invasive, physis sparing, and maintains an acceptable and painless reduction.

Many of these techniques do not respect physeal plates.^[2,6]^ Most operative methods need complementary 4 to 6 weeks of postoperative immobilization by casting.^[1–7]^

Current available techniques (modifications of Kirschner wiring or conventional elastic stable intramedullary nailing [ESIN]) have about the same rate of mild complications. Growth disturbance is a rare, but represents a very rare severe complication of transepiphyseal wire placement. Considering this fact and the drawback of the 4 to 6 weeks of cast immobilization of the conventional techniques, we looked for a solution which eliminates these problems.

In our report, we would like to introduce our modified ESIN method for operative treatment of severely displaced pediatric distal metaphyseal or metadiaphyseal radial fractures.

With 2 short and prebent, retrograde elastic titanium nails inserted proximal to the distal radial physis, a very stable stabilization can be achieved without the need for a prolonged period of cast immobilization. The nails do not cross the physeal plates, so the possibility of postoperative physeal arrest is reduced.

## 2 Patients and methods

We reviewed retrospectively 24 patients who underwent operations due to severely displaced distal forearm or diametaphyseal radial fracture between November 2012 and December 2015. All patients were treated with short double prebent intramedullary nails. Fifteen patients had isolated radial, and 9 children had associated ulnar fracture as well. There were 17 boys and 7 girls with an average age of 9.8 years (range, 6–16 years). The right hand was involved in 16 cases and the left hand in 8 cases. Indications for surgery included closed fractures with total radial or dorsal displacement and shortening. We excluded open fractures, physeal injuries, and pathological fractures. All procedures were performed under general anesthesia and C-arm image intensifier control. A single-shot antibiotic prophylaxis was routinely used. All children were treated by 3 surgeons experienced in the ESIN and percutaneous pinning technique as well. The parents were informed about other treatment options. We began immediately with a postoperative mobilization program and applied a short removable splint. The degree of anatomic reduction was confirmed on plain radiographs obtained at the first, fourth week, and sixth month postoperatively. Nails were removed 6 to 36 weeks after the operation in general anesthesia or in local anesthesia. Average follow-up was 17.8 months (range, 7–28 months).

Considering the fact that our method is a modification and special application of the conventional and existing ESIN technique, an ethical approval was not requested by the Ethical Committee of our institutions for this retrospective study. Clinical application of the modified technique has been accepted and permitted in 2009 by our medical review board by the Hungarian Pediatric Trauma Committee and by the Hungarian Pediatric Surgery Committee. Possible benefits, risks, and complications, along with other methods, were explained to all parents of each child, and an informed consent was obtained by them.

## 3 Operative technique

**Figure 1:**
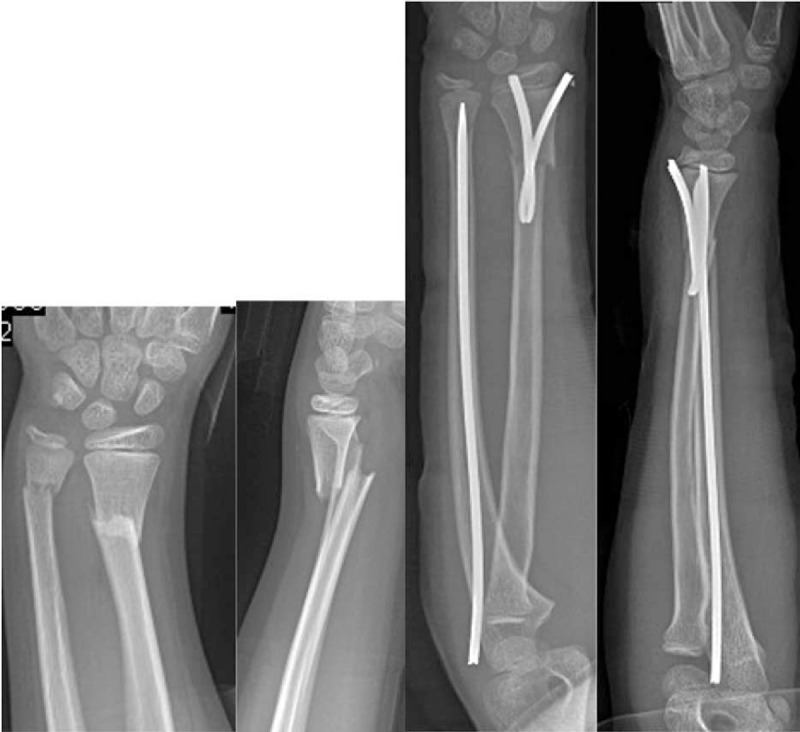
Pre- and postoperative x-rays of displaced distal forearm fracture.

**Figure 2:**
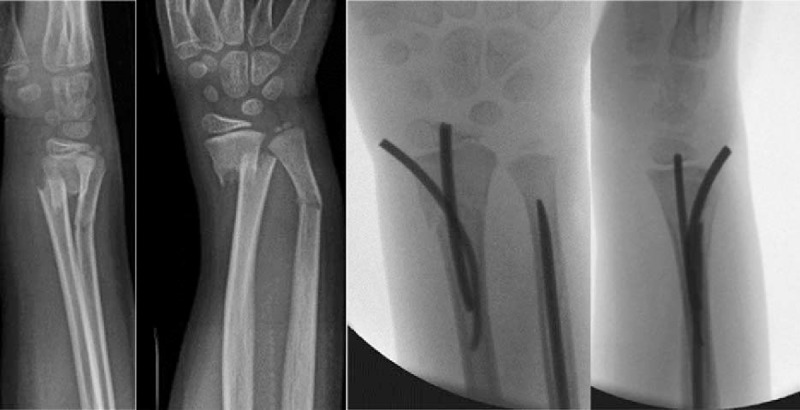
Pre- and postoperative x-rays of displaced distal forearm fracture.

**Figure 3:**
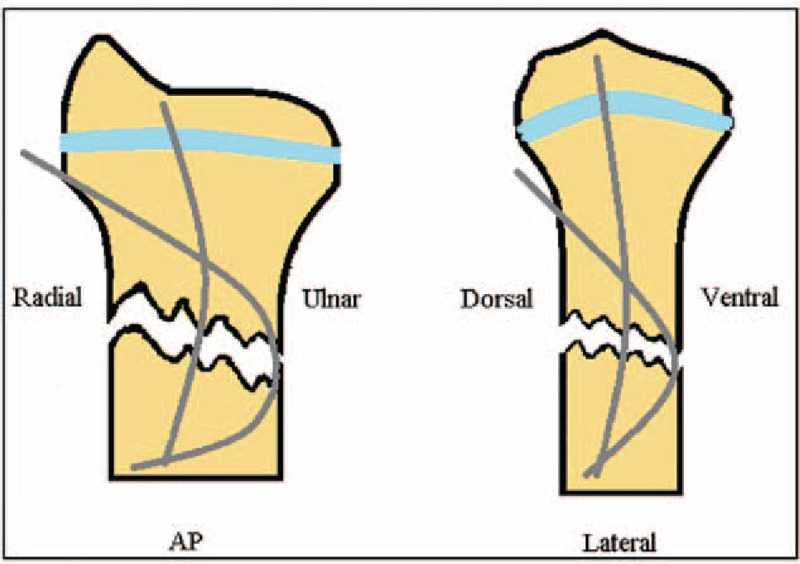
Position of the prebend short elastic nail is shown in the semantic picture.

Patients are under general anesthesia. The arm is extended and placed in a pronated position on a fluoroscopically translucent table. X-ray images of the lateral position and anteroposterior position before operation are shown in (Figs. 1 and 2). The first step is closed reduction under image intensifier. If reduction cannot be achieved by single manual manipulation a 2 to 3 mm diameter Kirschner wire (K-wire) or sharp elastic nail is inserted to the fracture gap from a dorsal stab incision. Using the pin as a lever arm, the distal fragment is raised up and slid forward onto the proximal end. After successful reduction, we determine the insertion points of the short elastic nails. The first is the dorsal site of distal radius, the area of Lister tubercle, just proximal to the physeal line of the radius. After skin incision, we gently dissect the soft tissues, and with an awl we open the medullary canal. A “C”-shaped prebent short and relatively thick (8–12 cm long and 2.5–3.5 mm diameter) titanium elastic nail is inserted into the distal medullary canal of the radius. The nail is gently moved forward along its curvature until its distal end enters the medullary canal of the proximal fragment. Then moderate force is applied, and the nail tip becomes impacted. In this position, the convex side of the nail faces the fracture line of the anterior cortex when observing from a lateral view. After insertion of the first nail, we determine the entry point for the second one. It is the distal radial site of the radius, just proximal to the growth plate. The second nail is usually thinner (2–2.5 mm in diameter) and also prebent to a “C”-form shape. Similarly to the technique described above, this nail is advanced forward along a radioulnar and distal to proximal curvature. After reaching the medullary canal of the proximal fragment, the nail is pushed further with a controlled force, until tightness is achieved. Observing from an AP-view, the convex side of the nail faces the ulnar cortex of the radius (Fig. 3). If the prebent curve of the short nail is not sufficient (the “C” shape is too flat), the end of the nail will get stuck in the ventral cortex. An overbent nail end, however, cannot be inserted into the proximal medullary canal. In this situation, the nail is pulled back, and the degree of the curvature is corrected. By pushing forward a malpositioned nail, an additional iatrogenic ventral or dorsal cortical fracture can occur, so it is forbidden to use uncontrolled force during nail insertion.

### 3.1 Postoperative care

The first 1 to 2 weeks, a short, removable splint is applied. Sport and hard physical activity is restricted for 6 weeks, but full range of movement can begin within a few days.

## 4 Results

Of the 24 patients, 3 presented irritation of the skin, which resolved after a relatively early removal of the radial nail. In the sixth postopertive week, all patients regained full range of motion. We have not observed any infection, tendon, nerve, or growth plate injury during the follow-up time. Average operational time was 19 minutes (range, 7–53 minutes). The necessary splinting time was reduced to an average 1 to 2 weeks. Usually, a short removable splint was sufficient for early pain-free mobilization. In the follow-up x-rays of the patients, 20 cases were considered as anatomic, 2 as good, and 2 as acceptable reduction. All the x-rays made 6 months postoperatively showed anatomic reduction, and there has been no sign of growth disturbance at the area of the distal radius.

## 5 Discussion

The gold-standard operative method for pediatric distal radial fracture is closed reduction and percutaneous pinning.^[2–8]^ Although the many variations of percutaneous pinning are simple and effective in unstable distal radial fractures, they bear many potential complications and disadvantages as well.^[8–12]^ Kirschner-wire-related complications are well known: migration of the pins, superficial infections, damage to the growth plate, skin irritation, and insufficient biomechanical ability to maintain the reduction without casting.^[8–10,13–15]^ This latter fact is the main drawback of this technique: in addition to the discomfort associated with operation, it is usually necessary to wear a long or short cast at least for 4 to 6 weeks postoperatively.^[2,3,5–9]^ Fractures in the area of the diametaphyseal junction represents a special problem: they are usually located too distally to be treated by classic ESIN and too proximal for conventional Kirschner wire fixation.^[2,6]^ Lieber et al^[2]^ used transepiphyseal pinning in these cases. Although the chance of a iatrogenic physeal injury is very small, a physeal arrest and progressive deformity can be a potential complication of any transepiphyseal stabilization.^[13–15]^ Others suggest a specially prebent long, physis-sparing elastic nail to eliminate this problem.^[16–18]^ All of these methods require postoperative plaster-cast immobilization.^[2,6,16–18]^ Analyzing our own cases of pediatric distal radial fractures treated by percutaneous pinning between 2008 and 2014 (n = 184), we found a total complication rate of 16.84% retrospectively. Superficial infection occurred in 8 children, skin irritation in 14, migration of the wires in 5 cases, unacceptable redislocation in 2 cases, and growth disturbance and postoperative deformity in 2 cases.

Using double elastic nailing, we have observed only mild complications like skin irritation which was caused by the end of the dorsally inserted and relatively too long nail under the skin. We hypothesize that this problem can be attributed to improper cutoff of nails during the learning curve period of the technique. When nails are cut in a maximally palmar-flexed position of the wrist just below the level of the skin, this irritation problem ceases.

Cutting off the nails outside the skin is also a possibility to eliminate this complication, although until now we sank each nails under the skin.

Anatomical reduction is not an absolute requirement treating these kinds of fractures due to the remodeling capacity of the distal radius. More severe dislocations (more than 30–40°) and older children (boys aged >12 years and girls aged >10 years) need reduction to the range of the remodeling capacity. K-wire osteosynthesis needs additional cast immobilization. Redislocation can occur even with properly applied casting and wiring because certain types of fractures (i.e., diametaphyseal configuration) are highly unstable. The x-ray results of our technique were all within the remodeling range, and they did not show even minimal tendency of redislocation. This indicates the superior stability of double elastic nailing to percutaneous pinning.

Our technique is a modification of the classic ESIN method. The biomechanical principles are the same as for long diaphyseal fractures, which are symmetrical splinting by 2 elastic nails, each supporting the inner cortex.^[19–23]^ The main difference is the length and the precontoured curve of the nails.

Ideally, the highest point of convexity of titanium elastic nails is at the level of the fracture symmetrically opposite each other.^[23]^ By long nails, it is directed toward the central region of the diaphysis, so in the cases of metaphyseal or diametaphyseal fracture, it would be very eccentric from the fracture line. Using short “C-shaped and mini” nails, the maximal curve is at the fracture, and the tension within the nail provides an optimal memory effect. The 2 nails ensure a long contact area with the inner cortex, which is mandatory for axial stability. Using 2 nails from different entry points, but from the same level, provides rotational, translational, and bending/bowing stability. We hypothesize that “short” ESIN nails inserted at the distal metaphyseal area works the same way as conventional ESIN in a shaft fracture. In an animal study, Johnson et al^[19]^ have shown that at 3 mm diameters or more beyond the fracture site, the length of the nail does not significantly affect the biomechanical properties of the construct.

We use thicker nails compared to conventional techniques. This is necessary because short nails have the greatest tensing effect in the broad diametaphyseal zone of the radius. For the second nail, we usually choose a smaller one because its main role is to add more rotational stability and thus we minimize the chance of a potential iatrogenic fracture. We have experienced differences in rotational stability using a thinner (2–2.5 mm) second nail, but insertion was easier. If we choose a thinner first nail (1.5–2 mm), flexion-extension stability significantly decreased. Stability was tested under intraoperative fluoroscopy in maximally dorsal-extended and palmar-flexed position of the hand. We achieved the best stability with combination of 2.5 to 3 and 2 to 2.5 mm nail diameters.

The insertion points (Lister tubercle and radiodorsal side of the radius) are the classic places for the insertion of conventional ESIN nails, located proximal to the physis, thus avoiding the possibility of iatrogenic growth plate injury. It is very important to visualize the dorsal and radial entry point adequately to avoid iatrogenic injury to the long extensor pollicis tendon and the superficial radial nerve. Incorrect insertion points or malpositions of the entry holes can jeopardize the physeal plates, the sensory branch of the radial nerve, or the tendons, which are also potential complications of the classic ESIN method.^[24–27]^ Advantages of our technique are the early mobilization with a short splint and the elimination of potential growth plate injury. We think that our technique is a minimally invasive, easily learnable alternative operative method, when compared to percutaneous pinning in metaphyseal, and plate or transepiphyseal osteosynthesis in cases of diametaphyseal-displaced fractures of the forearm of radius. Further prospective and biomechanical studies are required to verify our initial experience.
